# Complex Portal 2022: new curation frontiers

**DOI:** 10.1093/nar/gkab991

**Published:** 2021-10-29

**Authors:** Birgit H M Meldal, Livia Perfetto, Colin Combe, Tiago Lubiana, João Vitor Ferreira Cavalcante, Hema Bye-A-Jee, Andra Waagmeester, Noemi del-Toro, Anjali Shrivastava, Elisabeth Barrera, Edith Wong, Bernhard Mlecnik, Gabriela Bindea, Kalpana Panneerselvam, Egon Willighagen, Juri Rappsilber, Pablo Porras, Henning Hermjakob, Sandra Orchard

**Affiliations:** European Molecular Biology Laboratory, European Bioinformatics Institute (EMBL-EBI), Wellcome Genome Campus, Hinxton, Cambridge CB10 1SD, UK; European Molecular Biology Laboratory, European Bioinformatics Institute (EMBL-EBI), Wellcome Genome Campus, Hinxton, Cambridge CB10 1SD, UK; Fondazione Human Technopole, 20157 Milan, Italy; Wellcome Centre for Cell Biology, University of Edinburgh, Edinburgh EH9 3BF, UK; Department of Clinical and Toxicological Analyses, School of Pharmaceutical Sciences, University of São Paulo, Av. Professor Lineu Prestes 580, CEP 05508-000 São Paulo SP, Brasil; Bioinformatics Multidisciplinary Environment (BioME), Digital Metropolis Institute, Federal University of Rio Grande do Norte, Av. Odilon Gomes de Lima 1722, Capim Macio, 59078-400 Natal/RN, Brasil; European Molecular Biology Laboratory, European Bioinformatics Institute (EMBL-EBI), Wellcome Genome Campus, Hinxton, Cambridge CB10 1SD, UK; Micelio, Veltwijcklaan 305, 2180 Ekeren, Belgium; European Molecular Biology Laboratory, European Bioinformatics Institute (EMBL-EBI), Wellcome Genome Campus, Hinxton, Cambridge CB10 1SD, UK; European Molecular Biology Laboratory, European Bioinformatics Institute (EMBL-EBI), Wellcome Genome Campus, Hinxton, Cambridge CB10 1SD, UK; European Molecular Biology Laboratory, European Bioinformatics Institute (EMBL-EBI), Wellcome Genome Campus, Hinxton, Cambridge CB10 1SD, UK; Department of Genetics, School of Medicine, Stanford University, Palo Alto, CA, USA; Laboratory of Integrative Cancer Immunology, INSERM, 75006 Paris, France; Equipe Labellisée Ligue Contre le Cancer, 75006 Paris, France; Centre de Recherche des Cordeliers, Sorbonne Université, Université de Paris, 75006 Paris, France; Inovarion, 75005 Paris, France; Laboratory of Integrative Cancer Immunology, INSERM, 75006 Paris, France; Equipe Labellisée Ligue Contre le Cancer, 75006 Paris, France; Centre de Recherche des Cordeliers, Sorbonne Université, Université de Paris, 75006 Paris, France; European Molecular Biology Laboratory, European Bioinformatics Institute (EMBL-EBI), Wellcome Genome Campus, Hinxton, Cambridge CB10 1SD, UK; Dept of Bioinformatics - BiGCaT, NUTRIM, Maastricht University, Universiteitssingel 50, 6229 ER Maastricht, The Netherlands; Wellcome Centre for Cell Biology, University of Edinburgh, Edinburgh EH9 3BF, UK; Bioanalytics, Institute of Biotechnology, Technische Universität Berlin, 13355 Berlin, Germany; European Molecular Biology Laboratory, European Bioinformatics Institute (EMBL-EBI), Wellcome Genome Campus, Hinxton, Cambridge CB10 1SD, UK; European Molecular Biology Laboratory, European Bioinformatics Institute (EMBL-EBI), Wellcome Genome Campus, Hinxton, Cambridge CB10 1SD, UK; European Molecular Biology Laboratory, European Bioinformatics Institute (EMBL-EBI), Wellcome Genome Campus, Hinxton, Cambridge CB10 1SD, UK

## Abstract

The Complex Portal (www.ebi.ac.uk/complexportal) is a manually curated, encyclopaedic database of macromolecular complexes with known function from a range of model organisms. It summarizes complex composition, topology and function along with links to a large range of domain-specific resources (i.e. wwPDB, EMDB and Reactome). Since the last update in 2019, we have produced a first draft complexome for *Escherichia coli*, maintained and updated that of *Saccharomyces cerevisiae*, added over 40 coronavirus complexes and increased the human complexome to over 1100 complexes that include approximately 200 complexes that act as targets for viral proteins or are part of the immune system. The display of protein features in ComplexViewer has been improved and the participant table is now colour-coordinated with the nodes in ComplexViewer. Community collaboration has expanded, for example by contributing to an analysis of putative transcription cofactors and providing data accessible to semantic web tools through Wikidata which is now populated with manually curated Complex Portal content through a new bot. Our data license is now CC0 to encourage data reuse. Users are encouraged to get in touch, provide us with feedback and send curation requests through the ‘Support’ link.

## INTRODUCTION

Protein complexes, stable functional assemblies consisting of two or more associated polypeptide chains, are responsible for driving and regulating many cellular processes. Multi-chain assemblies perform many functions, including (a) positioning molecules involved in the same process in close proximity (b) bringing structure to disordered regions of proteins and (c) creating novel substrate binding sites at subunit interfaces. These assemblies can contain additional molecules, such as nucleic acids and small molecules. In budding yeast (1), around one in three proteins have a function in stable heteromeric complexes and in bacteria around one in five (see below).

Although the existence of many well-studied protein complexes has been recognized for decades, existing manually curated, species-specific catalogues were either not regularly maintained, e.g. CYGD (2) for yeast, or entries were curated based on individual papers rather than amalgamated knowledge, e.g. CORUM (3) for mammalian species. The Complex Portal (www.ebi.ac.uk/complexportal) was created to meet this unmet need: It is a manually curated, encyclopaedic resource that provides stable identifiers and summarizes compositional, topological and functional aspects of stable macromolecular complexes from a selection of model organisms and organisms of special interest. It enables protein complex identification in large-scale data analyses, contributing to the study of complex evolution and increasing our understanding of cell biology (4–8).

Since the last update (9), the coverage of model organism complexomes, the compendium of known complexes for a given species, has increased significantly, with that of *Saccharomyces cerevisiae* being maintained and added to as more experimental data leads to the identification of new assemblies (1) and the completion of a first draft of the *Escherichia coli* complexome. Work is now focused on annotating human complexes, wherever possible in collaboration with other data resources or scientific groups. We have also responded to the ongoing SARS-CoV-2 pandemic by creating the complexome of this organism and also of related viruses, in order to contribute to global efforts to respond to this threat.

**Figure 1.**
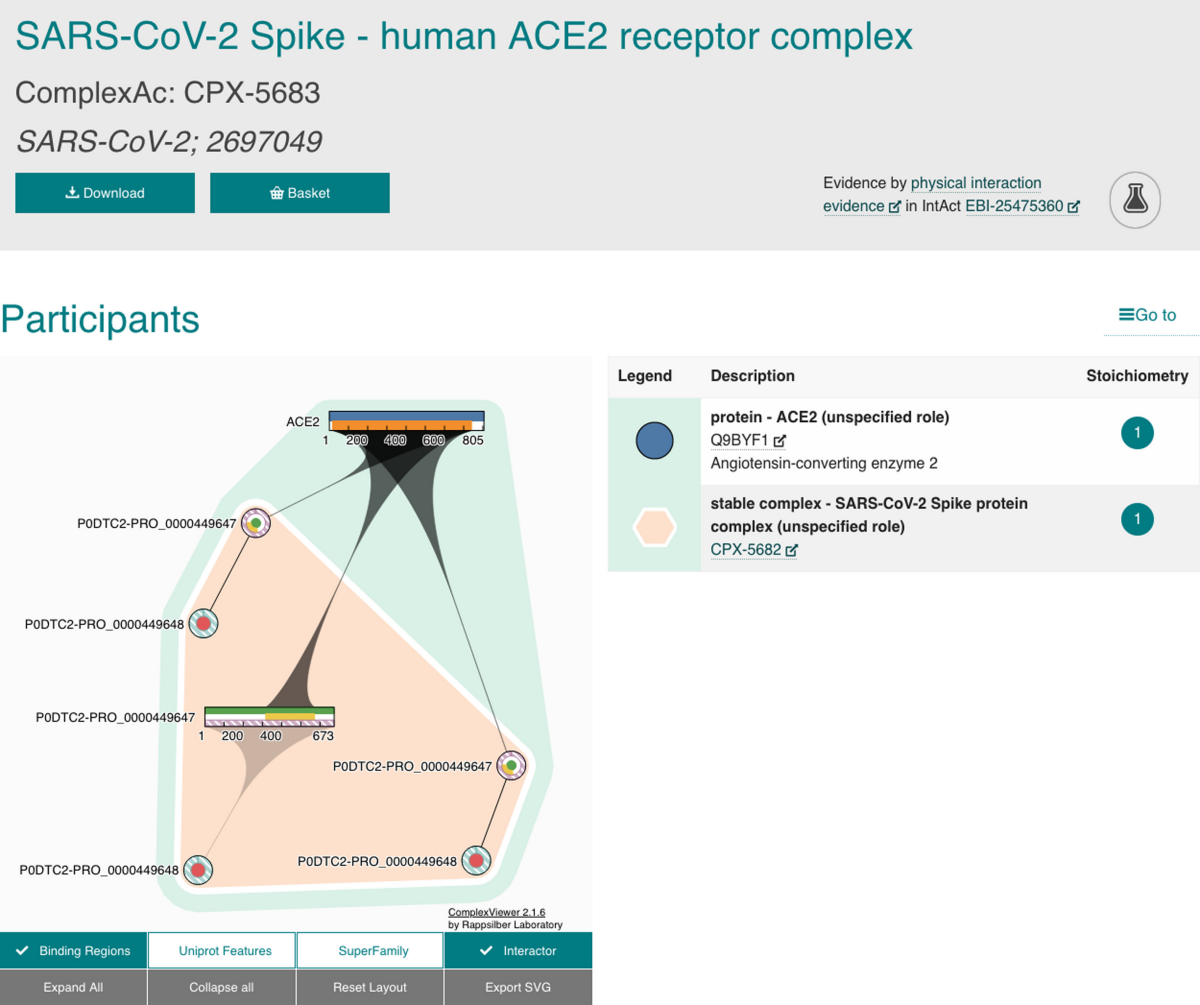
Top of the details page for SARS-CoV-2 Spike–human ACE2 complex displaying some of the new features available in the ComplexViewer (left side) and Participant table (right side).

We have improved ComplexViewer so that it can display multiple features simultaneously and show links between participants of sub-complexes and other complex participants. We updated the participant table so that all participants are now colour-coordinated between ComplexViewer and the table (Figure 1). We have made updates to the ComplexTab format, adding a ‘UniProt ID-only’ column that lists the UniProt accession numbers (and their stoichiometry) for the protein participants of complexes, including those that are part of subcomplexes and molecule sets.

We have expanded our community collaboration by contributing to a large-scale transcription cofactor analysis, by working with Wikidata contributors who have written a bot that populates Wikidata with Complex Portal content for semantic web reuse and by collaborating with the Cytoscape ClueGO App developers to incorporate Complex Portal entries as a new ontology for complex enrichment analyses. Finally, we changed our content license to CC0 to improve data accessibility and re-use.

## CONTENT

### Curation update

A protein complex is a functional, biological entity that contains two or more macromolecules (proteins, nucleic acids or small molecules) for which there is experimental or inferred evidence that these molecules stably interact with each other. As of release 241 (18 October 2021), 3572 complexes from 26 species have been curated and released. Each entry is species-specific, describing the complex composition, topology and function and linking out to external databases that provide further domain-specific information, such as structural details from wwPDB (10) or EMDB (11) or the role of the complex in metabolic reactions or signalling pathways in Reactome (12). Complex components are linked to primary reference resources; UniProt (13,14) for proteins, ChEBI (14) for small molecules and RNAcentral (15) for noncoding RNAs. Complexes that are also participants of larger complexes are linked to their own Complex Portal entries. In principle, we create separate entries for each compositional variant of a complex. However, some complexes, mainly the ribosomes, contain many participants that are potentially coded by two alternative, paralogous genes. In these cases, we create molecule sets, identified by identifiers of type EBI-{1–9}, containing the UniProt IDs of each of the paralogous proteins. On the website they appear as unlinked concatenations of gene symbols and species names, e.g. ‘rps4a_rps4b_yeast’.

Since our last update three years ago, we have focused on a number of curation priorities:

#### Escherichia coli complexome

**Figure 2.**
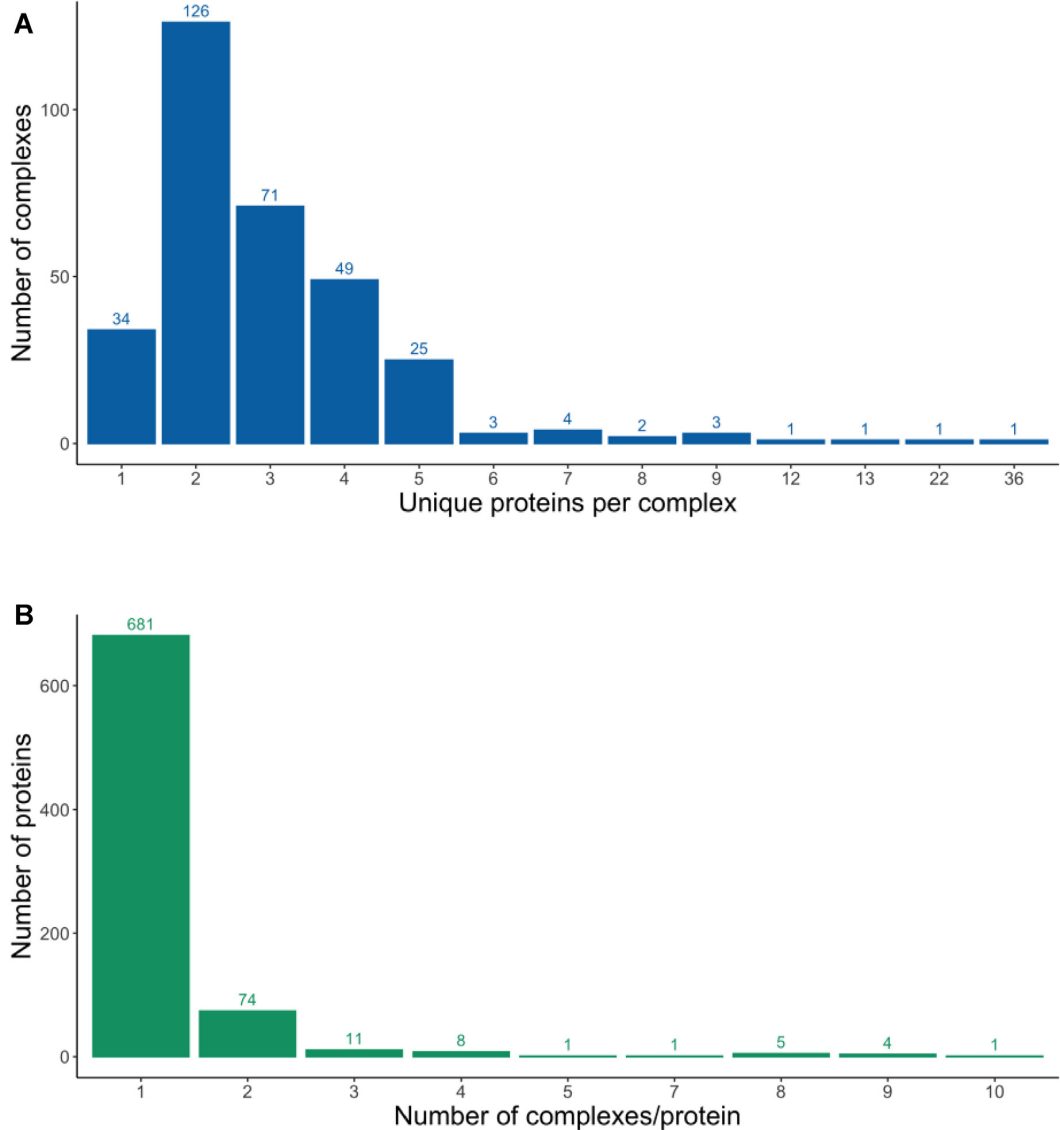
Most *E. coli* proteins are found in only one or two different complexes (**A**) and most *E. coli* complexes contain five or fewer unique proteins (**B**).

In December 2019, we released the first version of the *E. coli* K12 (NCBI reference taxon: 83333) complexome currently consisting of 321 complexes. This work was based on extensive literature mining and subsequent comparison with existing resources, in this case primarily UniProt KB and EcoCyc (16). This systematically annotated set of *E. coli* complexes includes, at the time of writing, 786 unique proteins (18% of the proteome). 95% (305) of complexes contain 5 or fewer proteins (median = 3) (Figure 2). 87% unique proteins (681/786) are found in only one complex and 9% protein (74/786) in two complexes with the remaining 31 proteins found in more than two complexes. This distribution is similar to what we saw for yeast (1) except that fewer proteins in general were found in heteromeric complexes (18% versus 32%).

As with the recently completed *S. cerevisiae* complexome, a watchlist of additional potential candidate complexes exists and these are being added to the dataset on an ongoing basis, if and when they are experimentally verified. Also, it must be recognised that *E. coli* K12 is a non-pathogenic laboratory strain and many proteins are cryptic or have been engineered out of the strain. Complexes containing such proteins are therefore absent from this model organism. For example, *E. coli* K12 does not express the PhnE permease due to the presence of an 8 bp insertion in *phnE* (17), therefore the phosphonate ABC transporter complex is not formed. To capture complexes that contain proteins not present in the K12 strain but that are essential for fully understanding the life-cycle of wild-type strains of these Gram-negative bacteria, the complex and its protein components are mapped to the species level for *E. coli*, NCBI taxon ID:562. Examples include the phosphonate ABC transporter complex (CPX-4382) and heat-labile enterotoxin IIB complex (CPX-2304).

#### Coronavirus complexes

In response to the COVID-19 pandemic we have curated coronavirus complexes as well as human targets of viral proteins (18). To date, we have released 21 SARS-CoV-2 complexes (taxon ID: 2697049), 16 SARS-CoV complexes (taxon ID: 694009) and 17 MERs-CoV complexes (taxon IDs: 1263720 and 1235996 [no reference proteome available]). They include mixed-species complexes of the viral Spike proteins with their host receptors ACE2 (Figure 1) and DPP4, respectively. As new experimental evidence emerges frequently for these complexes we have made use of our versioning protocol and updated complex components, stoichiometry and function. For example, new evidence from crystal and SAXS analyses led to an update of the stoichiometry of the nsp7-nsp8 primase (CPX-5690) from 8:8 to 2:2 while the function of the nsp10-nsp14 complex (CPX-5692) was updated from a guanine-N7 methyltransferase to a 3′-5′ exoribonuclease (GO:0000175). We continue to add new interaction evidence from IntAct (19) and structural evidence from wwPDB (20) and EMDB (21) at each release. These complexes are already used as cross-references by several external databases such as the IMEx resources (22), Gene Ontology (23,24), MatrixDB (25), Reactome, SIGNOR (26), UniProtKB and WikiPathways (27) thus allowing for a more integratable set of coronavirus-related information to be freely available to the research community.

#### Human complexes

Efforts are now focused on expanding the collection of manually curated human complexes. Exactly how many assemblies comprise the human complexome is a question still very much open to debate. We anticipate that most, but not all, of the intracellular complexes we have identified in *S. cerevisiae* are conserved in multicellular organisms, but human complexes often contain additional protein components or the existence of paralogous proteins lead to increased numbers of complex variants. One simple example of the latter case is the replication protein A complex, a single heterotrimer in yeast (CPX-21), but in humans one protein (P15927/Q13156) has been duplicated resulting in two heterotrimeric complex variants (CPX-1878/CPX-1879). Additionally, there will be an appreciable number of transmembrane and extra-cellular complexes with a role in the immune response, inter- and intracellular communication and signalling. A data mining exercise, undertaken in collaboration with the UniProt group, of information embedded in UniProtKB records has suggested that there may be at least 4000 different assemblies in the human complexome. To date, we have released 1255 human complexes including almost 200 that are either targets of viral proteins or play a role in the immune system, including the B-cell and T-cell receptor complexes, the interferon-receptor family of complexes and the complete complement cascade of the innate immune system. We are also focusing on dimeric transcription factor complexes, complementing a recent revision of the human transcription factor proteins undertaken by members of the Gene Ontology and Gene Regulation Consortia (28).

#### Linking chemistry to biology: enhancing enzyme annotation

Many enzymes are found in multi-chain complexes, which may serve to bring together multiple enzymes associated with a specific metabolic pathway, bring regulatory subunits in close proximity to the catalytic chain, enable the coordination of ligand binding, or create new binding sites in subunit interfaces. In order to improve our annotation of these assemblies, we are now adding cross-references to the Rhea knowledgebase of biochemical reactions (29) where it has been demonstrated that the enzyme has this activity in the context of a given complex. Rhea uses the chemical ontology ChEBI to describe reaction participants, their chemical structures and chemical transformations and enables a more granular description of the enzyme/substrate interaction. For example, human serine palmitoyltransferase exists as four variant complexes (CPX-6663, CPX-6664, CPX-6665, CPX-6681) which share a common E.C. number (2.3.1.50). However, the substrate specificities of these complexes vary, due to the positioning of side chain residues of differing amino acids in the alternate complex components, and this can be fully described by the addition of the appropriate Rhea cross-references. Rhea cross-references are currently available in download files, and will be visualised on the website in the near future.

Enzymes are not the only complex components which bind small molecules. Rhea encompasses transporter activity within its concept of a reaction, and cross-references are increasingly being added to complexes involved in transportation. The ligands that are bound by receptors are also systematically captured as a ‘ligand’ annotation topic and are described by both a recommended, human-readable protein, peptide or small molecule name and a UniProtKB or ChEBI accession number, as appropriate.

#### Defining curation practice for obsoleting complexes and versioning

When the research community's understanding of a complex's existence changes to the extent that we need to delete an existing entry, or we decide to merge a sub-complex into a larger assembly, the original entries remain available in previous release files accessible via our ftp repository (http://ftp.ebi.ac.uk/pub/databases/intact/complex/). If a complex has been merged into an existing entry, the accession number of the obsoleted complex is added to the complex it has been merged into as a secondary identifier, allowing an external user to still retrieve an entry. Secondary identifiers are available in all download files, while the website currently only displays the primary accession number (e.g. CPX-3042 is now part of CPX-2161). This enables us to adhere to the FAIR principle of data Findability. More minor updates, for example the identification of an additional participant, are indicated by entry versioning, as previously described (1,9).

## WEBSITE SEARCH AND DATA VISUALIZATION

### ComplexViewer and participant table

We have made a number of improvements to the ComplexViewer (3) (Figure 1):

- *Zooming into a protein sequence*: clicking on a protein icon expands it into a short sequence bar. Clicking into the bar brings up a pop-up menu from which the sequence can be expanded further to four different zoom levels or collapsed again into the original circular icon. This feature is now available on touchscreens.
- *Subcomplexes*: if a complex has another complex as a participant, all proteins are now displayed and grouped by subcomplex membership using differently coloured backgrounds for each subcomplex (e.g. CPX-1556).
- *Binding features in subcomplexes:* binding features are displayed between any participants in a complex, which now includes participants which are part of a subcomplex and bind another molecule outside the subcomplex, e.g. the binding of human ACE2 receptor to the SARS-CoV-2 Spike S1 protein chain (CPX-5683) (Figure 1).
- *Undefined binding regions:* are now represented as full length features of the participant and the range is hatched, while defined ranges are filled solidly (e.g. CPX-2158).
- *Multiple feature types and ranges:* are displayed as separate tracks in the bar representation of a protein and separate wedges or circles within the circular icon (e.g. CPX-1919 or CPX-1003). Different feature types can be turned on and off using a new set of buttons below the viewer window.

Additionally, the participant table is now colour-coordinated, matching the node colours of proteins or background colours of complexes as participants.

### Website updates

We have refreshed and updated the Home, About and Documentation pages, including adding more information about our curation practices, handling of edge cases and documentation about our file formats. These new pages are also linked to our GitHub repository which allows us to make any updates easier and quicker.

## DATA ACCESS OPTIONS

### ComplexTab

Complexes may contain molecules other than proteins, such as nucleic acids or small molecules, but some users, for example mass spectrometry proteomics scientists, are only interested in the protein components. Protein complexes can be subcomponents of larger assemblies, and users previously needed to parse the files separately to retrieve the participant of these subcomplexes. In response to requests from this user community, we have added a new column to the tab-delimited format, ComplexTab, that contains a list of UniProt accession numbers (including isoform or post-processes chain extensions) in pipe-separated style with stoichiometry in parentheses.

### Data licencing

To ensure our data is available for reuse by all interested parties, and in line with EMBL-EBI policy, our licence has been updated to Creative Commons Public Domain (CC0) License (https://creativecommons.org/publicdomain/zero/1.0/). This applies to all Complex Portal data, i.e. PSI-MI XML3.0 (30), MI-JSON and ComplexTab files (9), as well as data directly accessed via web pages/services.

## COLLABORATION AND COMMUNITY INVOLVEMENT

### Identification of putative transcription cofactor complexes through the Gene Regulation Ensemble Effort for the Knowledge Commons (GREEKC) collaboration

The GREEKc collaboration was an EC-funded COST action aimed at integrating data and knowledge pertaining to gene regulation, of which the Complex Portal was an active participant throughout. As part of this effort, Velthuijs *et al.* (31) have used the Complex Portal as a verification dataset in their analysis of potential transcription cofactors. By combining inferred complexes from hu.MAP 2.0 (32), curated complexes from CORUM (33) and curated physical interaction data from IntAct [this volume NAR paper] and BioGrid (34) with a selected set of transcription-related Gene Ontology terms we have identified more than 1500 putative transcription cofactors. 415 of these are already participants of complexes in Complex Portal, and the remaining proteins will be curated into Complex Portal if they are identified as components of complexes.

### Integration of complexes into Wikidata

Wikidata (https://www.wikidata.org/) is part of the infrastructure provided by the Wikimedia Foundation and a sister project of Wikipedia; it initially provided a semantic web infrastructure for encyclopedic knowledge to be used in Wikipedia (35), but has since gained traction as a generic linked open resource; over the past years a number of life sciences resources have been aligned to Wikidata, including subsets of UniProt, the Gene Ontology and ChEBI.

**Figure 3.**
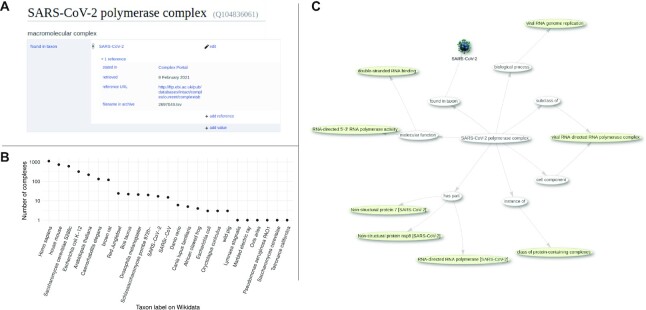
Complex Portal and Wikidata. (**A**) example of an entry assertion in Wikidata with provenance pointing to Complex Portal (Q104836061). (**B**) Number of protein complexes in Wikidata per taxon (https://w.wiki/3ggX). (**C**) Subset of Wikidata connected to the SARS-CoV-2 polymerase complex (https://w.wiki/3eta)

After we released the first eleven SARS-CoV-2 complexes in April 2020 we joined the COVID19 Virtual Elixir BioHackathon 2020 (https://github.com/virtual-biohackathons/covid-19-bh20/wiki). During this hackathon we added Wikidata entities for these eleven complexes using a semi-automated curation pipeline using OpenRefine (https://openrefine.org/) and the Wikidata Integrator Python module (36). Through this integration into Wikidata, Complex Portal identifiers (https://www.wikidata.org/wiki/Property:P7718) for SARS-CoV-2 complexes as well as selected human complexes, which had already been manually added to Wikidata, were immediately used in the WikiPathways COVID-19 Pathways Collection (http://covid.wikipathways.org/) (36) which is part of the COVID-19 Disease Map project (https://covid19map.elixir-luxembourg.org/minerva/) (37) that also includes the Reactome COVID-19 project. As an extension to this initial SARS-CoV-2-focused collaboration we have subsequently developed a Wikidata Complex Bot, reconciling the entries in Complex Portal to Wikidata. The Complex Bot parses the Complex Portal releases and enriches the Wikidata environment, connecting proteins by their common presence in complexes, linking the new entries to existing entries and matching GO complexes with their Wikidata IDs. As a result, Complex Portal data is now available on the semantic web and updated regularly in line with our regular data releases and integrated with related UniProt, GO and ChEBI data in semantic web format (Figure 3). This enables rapid integration with other linked-data sources using Wikidata as a proxy. To be able to run bots on Wikidata a bot-flag was requested (https://www.wikidata.org/wiki/Wikidata:Requests_for_permissions/Bot/ComplexPortalBot) and approved after an online vetting process, where the rationale and the software was assessed. The open source code of the bot can be found at https://github.com/lubianat/complex_bot. Currently, the bot runs manually; after each Complex Portal update a request is sent to the bot developer to run the bot on the recent update. We are working towards an automatic integration of the two processes, similar to the continuous integration applied elsewhere (36). Another use of the Complex Portal data in Wikidata is the visualisation of complex information with Scholia (38). For example, https://scholia.toolforge.org/complex/Q104836061 shows information for the SARS-CoV-2 polymerase complex (CPX-5742). Similarly, protein pages show in which complexes they participate (e.g. https://scholia.toolforge.org/protein/Q90038963 for SARS-CoV-2 NSP7).

### Additional usage and collaborations

Complex Portal identifiers are already being used as the preferred identifiers for complex entities in, among others, IMEx, Gene Ontology, *Saccharomyces* Genome Database (39) and SIGNOR curation efforts and more recently for causal interaction curation efforts based on MI2CAST curation guidelines (40), WikiPathways (27) annotations (via the BridgeDb (41) mapping service) and the COVID-19 Disease Map project (37). The use of Complex Portal identifiers in WikiPathways was enabled by manually creating complex entities in Wikidata with cross-references to Complex Portal, an effort that preceded and initiated our Wikidata bot development. An ongoing collaboration with the UniProt team will drive further work on the human complexome and an enhanced import of data from the Complex Portal into UniProt records is under active discussion.

Complex Portal data, together with molecular interaction data from IntAct, Reactome and SIGNOR, is integrated into the Open Targets (42) partnership that uses human genetics and genomics data for systematic drug target identification and prioritisation, via our bespoke graph database. Additionally, we provide a JSON file from the same graph database, containing protein-to-complex mappings.

### Functional analysis through ClueGO

**Figure 4.**
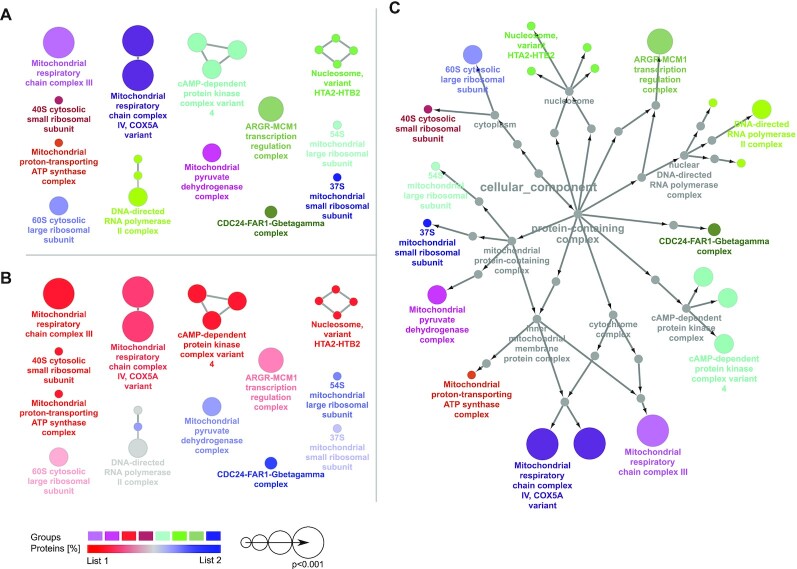
Output of an enrichment analysis for Complex Portal complexes of *S. cerevisiae* proteins using ClueGO. Two different lists of proteins were compared. (**A**) functionally grouped complexes, (**B**) distribution (%) of the proteins from list 1 (red) and 2 (blue) within each of the complexes from (A), (**C**) relationship of complexes based on the Cellular Component part of the Gene Ontology Tree.

The ClueGO App (43) is a powerful platform for functional enrichment analysis within Cytoscape (44). Bespoke Complex Portal ontology files have been created for a selected number of species with the Complex Portal complexes being leaf nodes of the Gene Ontology Cellular Component class. These new ontology files are available starting with ClueGO version 2.5.8 and allow users to conduct enrichment analyses for complex composition, see Figure 4. We will further collaborate with the ClueGO team to extend the Complex Portal Ontology, and to create new visualizations for Complex Portal data.

## SUMMARY AND FUTURE PLANS

With the completion of the draft complexomes for *S. cerevisiae* and *E. coli* we are now fully focusing on completing a first draft of the human complexome. We are looking to extend our collaborations with other resources to increase our coverage of other model organisms, a paradigm successfully initiated with *Saccharomyces cerevisiae* (39). We are currently focusing on immune system complexes through collaborations with WikiPathways, the COVID-19 Disease Map project and the Cellxgene initiative (45).

We are developing an import pipeline for heteromeric structures from PDBe which will speed up the manual part of the curation process by populating all standardized, structured fields directly from the PDB files. We are also working with the group of Colin Logie (https://molbio.science.ru.nl/about/molecular-biology/colin-logie/) who is studying the relation between chromatin structure and transcription and is providing extensive lists of important curation targets. The group is developing a pipeline to identify additional potential protein complexes with a role in cotranscription which will be further evaluated by manual curation in the Complex Portal.

We actively encourage curation requests and user feedback which will improve our databases and services. Please contact the Molecular Interaction Team via our support page at https://www.ebi.ac.uk/support/complexportal. Information about curation is provided at https://www.ebi.ac.uk/complexportal/documentation. Extensive training material on how to best use our resource is available at https://bit.ly/Complex-Portal-training.

## DATA AVAILABILITY

The Complex Portal is a community project. Developers can contribute to the code at https://github.com/Complex-Portal/complex-portal-view.

Data can be accessed either via our ftp site (ftp.ebi.ac.uk/pub/databases/intact/complex/current/) or our REST API (https://www.ebi.ac.uk/intact/complex-ws/).

Lists of putative complexes on our ‘watch lists’ are available on request (https://www.ebi.ac.uk/support/complexportal).

Wikidata Integrator python module: https://github.com/SuLab/WikidataIntegrator.

Wikidata bot: https://github.com/lubianat/complex_bot.
